# Acidic Mesoporous Zeolite ZSM-5 Supported Cu Catalyst with Good Catalytic Performance in the Hydroxysulfurization of Styrenes with Disulfides

**DOI:** 10.3390/nano7120459

**Published:** 2017-12-19

**Authors:** Jun Hu, Chaojie Zhu, Feifei Xia, Zhongxue Fang, Fengli Yang, Jushi Weng, Pengfei Yao, Chunzhi Zheng, Hai Dong, Wenqian Fu

**Affiliations:** 1 School of Chemical and Environmental Engineering, Jiangsu University of Technology, Changzhou 213001, China; jhu@jsut.edu.cn (J.H.); xff7461198@163.com (F.X.); 252391742@163.com (F.Y.); wengjushi@jsut.edu.cn (J.W.); yaopengfei@jsut.edu.cn (P.Y.); zhengcz@jsut.edu.cn (C.Z.); 2 School of Petrochemical Engineering, Changzhou University, Changzhou 213164, China; zhcj1103@163.com (C.Z.); fangzhongxue@cczu.edu.cn (Z.F.); dh@wxtcxny.com (H.D.)

**Keywords:** mesoporous zeolite ZSM-5, copper, hydroxysulfurization, acidity

## Abstract

Development of highly active heterogeneous catalysts is an effective strategy for modern organic synthesis chemistry. In this work, acidic mesoporous zeolite ZSM-5 (HZSM-5-M), acidic-free mesoporous zeolite TS-1 (TS-1-M), and basic ETS-10 zeolite supported metal Cu catalysts were prepared to investigate their catalytic performances in the hydroxysulfurization of styrenes with diaryl disulfides. The effect of pore size and acidities of the supports, as well as the Cu species electronic properties of the catalysts on reaction activity were investigated. The results show that Cu^+^ and Cu^2+^ binded on HZSM-5-M show the highest activity and product selectivity for the desired *β*-hydroxysulfides compounds.

## 1. Introduction

*β*-Hydroxysulfides are important intermediates for synthesis of various valuable biological active compounds, pharmaceuticals, and natural products, such as renin and leukotrienes [1,2,3]. Traditional methods for the preparation of *β*-hydroxysulfides involve the ring opening of epoxides with sulfenyl that comes from thiols in the homogenous metal-catalyzed reaction systems [4,5,6]. However, this protocol usually suffers from relatively low activity and undesirable side reaction [1]. To improve the reaction activity and product selectivity, many strategies have been developed. For example, various functional organic ligands were added into reaction mixtures for modification of the metals’ electronic property to improve the reaction activity [7,8]; irradiation or peroxides were used in the reaction systems for the generation of sulfenyl radicals, improving the product selectivity [3,9,10]. Nevertheless, the product separation from the reaction mixture is complicated and catalyst reuse is difficult. From a sustainable and practical point of view, developing a highly efficient heterogeneous catalyst is significant for preparation of *β*-hydroxysulfide compounds.

It is well known that crystalline aluminosilicate zeolites have high surface area, tunable acidity, and unique framework structure [11], which as supports or catalysts show superior catalytic performance in many organic syntheses, such as carbon–carbon coupling [12], Knoevenagel [13], and oxidative coupling reactions [14]. As a continuous work focus on development of highly efficient heterogeneous catalyst for synthetic chemistry, in this work, H-form mesoporous zeolite ZSM-5 (HZSM-5-M) supported Cu catalyst (Cu/HZSM-5-M) was prepared and applied in the hydroxysulfurization of alkenes. The results show that Cu/HZSM-5-M exhibits high activity and good product selectivity, and shows no significant change in catalytic activity after seven repetitions.

## 2. Experimental Section

### 2.1. Material Synthesis

Water glass is a sodium silicate (Na_2_O∙4SiO_2_, SiO_2_ 24.6 wt. %, Na_2_O 6.8 wt. %) solution, which was purchased from Zhejiang Tongxiang Water Glass Factory (Tongxiang, China). The other chemicals, aluminum sulfate (Al_2_(SO_4_)_3_∙18H_2_O, 99%) and tetrapropylammonium hydroxide (TPAOH, 25%), were purchased from Sinopharm Chemical Reagent Co., Ltd. (Shanghai, China).

Mesoporous zeolite ZSM-5 (ZSM-5-M) was synthesized in the gel containing cationic copolymer (denoted as RCC) as mesoscale template, where RCC was synthesized from diallylamine and diallylammonium chloride [15]. As a typical run, 15.8 mL water glass, 20 mL water and 0.4 mL tetrapropylammonium hydroxide (25 wt. %, TPAOH), and 6 mL RCC was mixed in sequence and stirred for 2 h. Next, 40 mL acidic aluminum sulfate solution (0.05 mol/L) was added dropwise in to the mixture and stirred for 2 h. The composition of this gel was Al_2_O_3_/45SiO_2_/12Na_2_O/0.28RCC/0.2TPAOH/2049H_2_O. After this gel hydrothermal treatment at 170 °C for 48 h in Teflon-coated stainless-steel autoclave, the sample was collected by filtrating, washing with water, drying at 100 °C for 12 h, and calcination at 550 °C for 5 h in air. Micropore zeolite ZSM-5 was synthesized with same procedure in the absence of RCC. Mesoporous zeolite TS-1 (TS-1-M) and ETS-10 were prepared according to previous works [13,16]. ZSM-5-M and ZSM-5 samples were ion-exchanged with NH_4_NO_3_ (1 M) solution for 4 h at 80 °C, and was collected by filtration, drying at 100 °C for 12 h, and calcination in air at 500 °C for 4 h for transformation to H-form samples (HZSM-5-M and HZSM-5).

### 2.2. Catalyst Preparation

The catalyst with Cu metal loading of 3 wt. % was prepared from incipient-wetness impregnation method. Typically, HZSM-5-M was impregnated with a solution containing an appropriate amount of Cu(NO_3_)_2_∙6H_2_O, followed by drying at atmospheric temperature for 24 h and at 100 °C for 12 h. Finally, the dried sample was calcined at 450 °C for 4 h in air, and the obtained catalyst sample was denoted as Cu/HZSM-5-M. The Cu/HZSM-5, Cu/ETS-10, and Cu/TS-1-M catalysts were prepared using the same method. Additionally, Fe and Co supported on HZSM-5-M (Fe/HZSM-5-M and Co/HZSM-5-M) catalysts were also prepared by the same method except for using iron nitrate hydrate and cobalt nitrate hydrate, respectively.

### 2.3. Characterization

X-ray powder diffraction (XRD) pattern was obtained on a D/MAX 2500/PC powder diffractometer (Rigaku, Ltd., Tokyo, Japan) by using a Cu *Kα* radiation source. N_2_ adsorption-desorption isotherms were measured at −196 °C on a Micromeritics ASAP2020M apparatus (Micromeritics Instrument Ltd., Darmstadt, Germany). The acidities of the supports were measured by ammonia temperature-programmed desorption (NH_3_-TPD) on a Micromeritics ASAP2920 instrument (Micromeritics Instrument Ltd., Darmstadt, Germany) according to previous work [15]. Total acidic site density of the support was determined by acid-base titration method [15]. The obtained NH_3_-TPD curve was deconvoluted at different maximum peak temperatures with a Gaussian function for fitting [17], and the peak areas were calculated. In this manner, the peak areas were correlated with the amount of adsorbed NH_3_ in different temperature regions. The morphology and mesoporous structure of ZSM-5-M were observed by field-emission scanning electron microscope (SEM, SUPRA55, Zeiss Gruppe, Heidenheim, Germany) and transmission electron microscope (TEM, JEM-2100, JEOL Ltd., Tokyo, Japan), respectively. The electronic state of the Cu species in catalysts was analyzed by X-ray photoelectron spectroscopy (XPS) on an ESCALAB MK II system (VG Scientific Ltd., East Grinstead, UK). Cu content and atomic Si/Al(Ti) in the different samples were determined by inductively coupled plasma optical emission spectroscopy (ICP-OES) on a Perkin-Elmer 3300DV emission spectrometer (PerkinElmer, Waltham, MA, USA). The Cu content in Cu/ZSM-5-M, Cu/ZSM-5, Cu/TS-1-M, and Cu/ETS-10 catalysts was 3.0, 2.9, 3.0, and 2.9 wt. %, respectively (Table S1).

## 3. Results and Discussion

### 3.1. Catalyst Characterization

**Figure 1:**
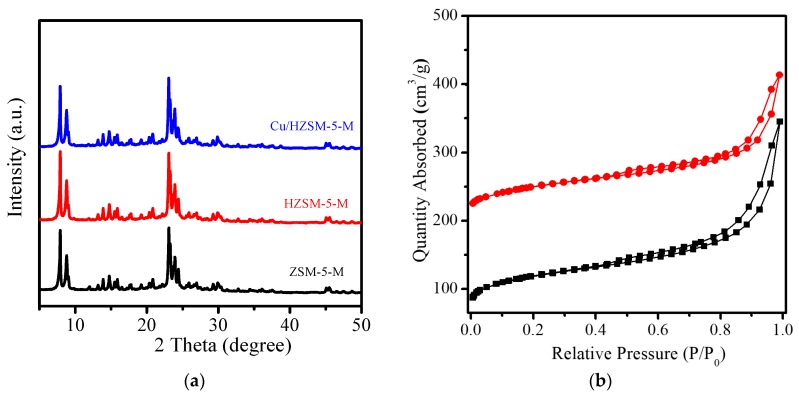
(**a**) XRD patterns of ZSM-5-M, HZSM-5-M and Cu/HZSM-5-M samples; (**b**) N_2_ adsorption isotherms of the (■) ZSM-5-M and (●) Cu/HZSM-5-M samples.

XRD patterns of ZSM-5-M and HZSM-5-M in Figure 1a give diffraction peaks at 2*θ* = 7.9°, 8.9°, 23°, 23.9°, and 24.5°, which is typical characteristics of ZSM-5 zeolite structure [15]. After loading of Cu on HZSM-5-M and calcination, the XRD diffraction peak position and intensity did not change, indicating that the ZSM-5 zeolite structure is well maintained for the Cu/HZSM-5-M sample. The diffraction peaks related with Cu species are not observed in the XRD pattern of Cu/HZSM-5-M, suggesting that small metal particles are dispersed in the Cu/HZSM-5-M sample. Similar phenomena were also observed in XRD patterns of TS-1-M and basic ETS-10 supported Cu catalysts (Figure S1). Nitrogen adsorption-desorption isotherms of ZSM-5-M and Cu/HZSM-5-M samples give a steep step at relative pressure of 0.6–0.96 (Figure 1b), which is related to the presence of mesopores in the samples. Their corresponding mesopore size mainly centered at 22 and 21 nm (Figure S2), respectively.

**Figure 2:**
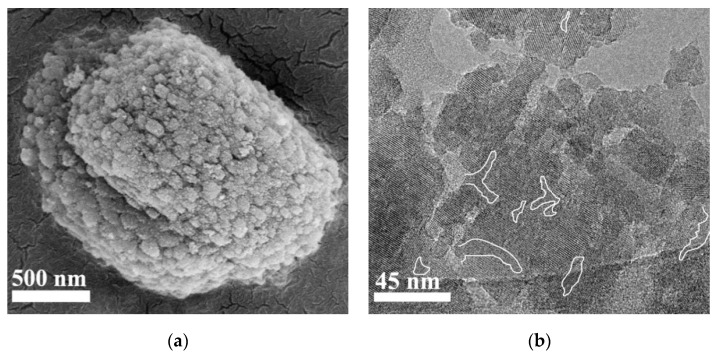
(**a**) Scanning electron microscope (SEM) and (**b**) transmission electron microscope (TEM) images of the ZSM-5-M sample.

Figure 2a shows the SEM image of ZSM-5-M, giving a cylinder-like morphology with rough surface. The large cylinder-like particles are composed of nano-crystals with sizes of 50–150 nm. In this case, meso-macropores could be formed in the nanocrystal assemblies. The morphology of the TS-1-M, ZSM-5, and ETS-10 zeolites were also observed by SEM technique, and the results are shown in Figure S3. The SEM image reveals that the TS-1-M crystal particle exhibits a rough surface and has a particle size of 200–300 nm, while both of ZSM-5 and ETS-10 zeolite have a smooth surface with a large particle size.

The porous structure of the ZSM-5-M was characterized by TEM technique. It was observed that not only many intra-crystalline mesopores (white lines), but also abundant inter-crystalline mesopore and marcopores (light areas), are present in ZSM-5-M particles (Figure 2b).

**Figure 3:**
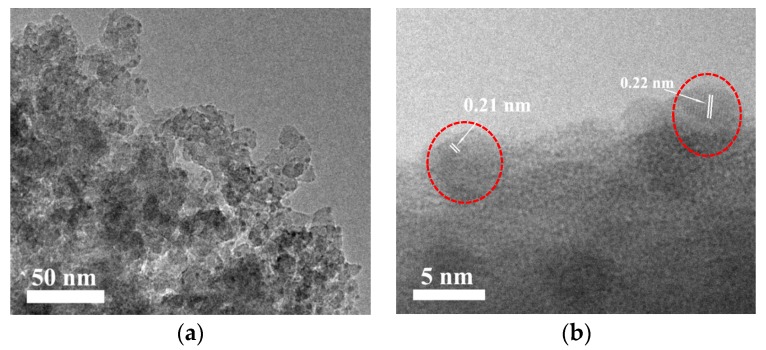
TEM images of the Cu/HZSM-5-M sample (**a**) in a low-magnification and (**b**) in a high high-magnification (The metal particles in Figure 3b were surrounded by red circle).

The dispersion of metal partials on Cu/HZSM-5-M was observed by TEM technique, and the results are shown in Figure 3. The low-magnification TEM image shows that small metal particles (3–5 nm) highly disperse on the Cu/HZSM-5-M (Figure 3a). The high-magnification TEM image in Figure 3b displays that some metal particles with a lattice fringe of 0.21 and 0.22 nm corresponds to the (111) crystal plane of Cu_2_O and (200) crystal plane of CuO [18], respectively. This result indicates that some copper could be present in the form of Cu_2_O and CuO on the Cu/HZSM-5-M sample.

**Figure 4:**
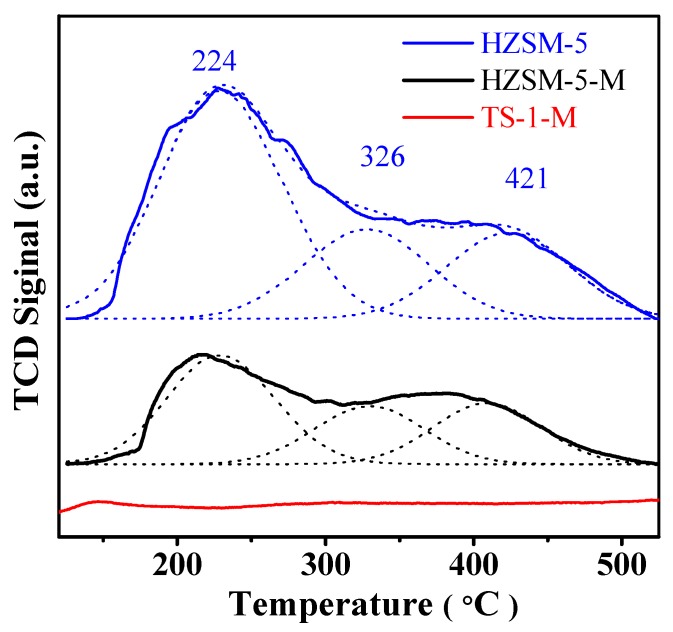
NH_3_-TPD curves and Gaussian deconvoluted peak of the samples.

Figure 4 shows the NH_3_-TPD curves of the HZSM-5 and HZSM-5-H, giving similar NH_3_ desorption profile with peaks around 224, 326, and 421 °C, indicating that weak, medium, and strong acid sites are present in these samples. However, HZSM-5 has a large number of total acid site densities relative to HZSM-5-M. The acid-base titration results also demonstrated that the number of weak, medium and strong acid site on HZSM-5 is higher than that on HZSM-5-M (Table 1). The NH_3_ desorption signal of TS-1-M sample was inconspicuous, indicating the absence of acidity on TS-1-M sample.

**Figure 5:**
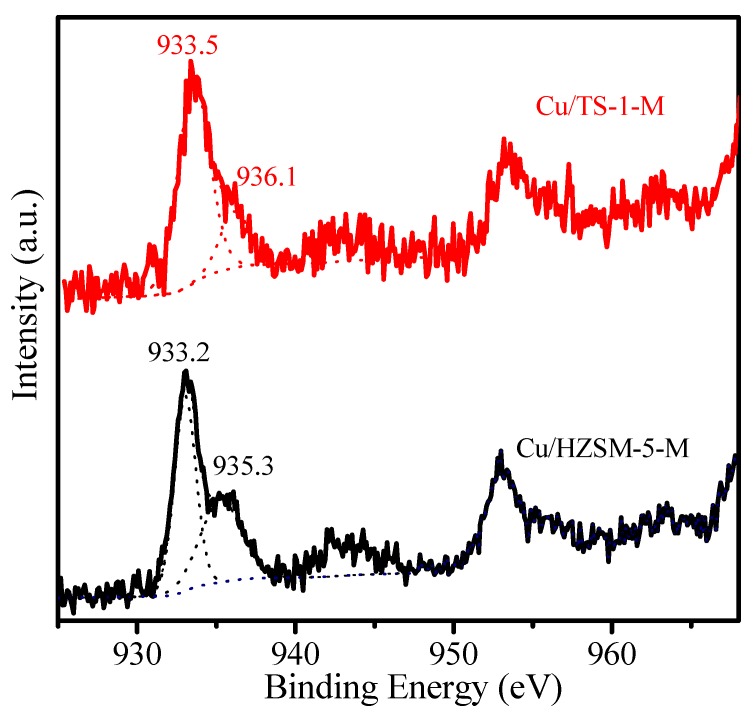
XPS spectra Cu2p_3/2_ of the Cu/HZSM-5-M and Cu/TS-1-M samples.

The chemical state of the Cu species on Cu/HZSM-5-M and Cu/TS-1-M catalysts was investigated by XPS. The Cu2p_3/2_ spectra in Figure 5 show two binding energies approximate 933.2 and 935.3 eV on Cu/HZSM-5-M catalyst corresponding to the Cu^+^ and Cu^2+^ species, respectively [19]. A similar phenomenon also existed on the Cu/TS-1-M catalyst. Meanwhile, the binding energies of the Cu^+^ and Cu^2+^ species on the Cu/HZSM-5-M catalyst are lower than those on Cu/TS-1-M catalyst. This could be due to the fact that the metal-support interaction on the Cu/HZSM-5-M is stronger than Cu/TS-1-M. In addition, the content of the Cu^+^ species is higher than Cu^2+^ species on both of Cu/HZSM-5-M and Cu/TS-1-M catalysts by XPS analysis (Table S2).

### 3.2. Catalytic Performance

To optimize the reaction conditions, the hydroxysulfurization of styrene with diaryl disulfides was carried out with various oxidants and solvents under different temperatures (Table S3). Preliminary solvent screening experimental results show that dimethylsulfoxide (DMSO)/H_2_O was the effective mixture solvent in the presence of iodine as oxidant at 80 °C, affording styrene conversion of 89% and 1-phenyl-2-(phenylthio)ethanol product selectivity of 63% (entries 1–6). After designation of DMSO/H_2_O as solvent, the influence of oxidant on reaction activity was investigated. Compared to *t*-butylhydroperoxide (TBHP) and H_2_O_2_, iodine proved to be the most efficient oxidant (entries 7 and 8). When using O_2_, K_2_S_2_O_8_, and KMnO_4_ as oxidants, this reaction did not occur (entries 9–11). By optimization of the reaction temperature, it was found that the highest styrene conversion and the product selectivity at 80 °C were obtained (entries 12–15). Consequently, the reaction conditions were determined and followed as: styrene (1.0 mmol), diaryl disulfides (0.6 mmol), 0.2 mmol iodine, DMSO/H_2_O (1:1) as solvent, temperature of 80 °C. The good reaction activity was obtained by using iodine as an oxidant, which could be due to fact that iodine can promote the formation of sulfonium ions from dibenzyl disulfide [20].

The catalytic performance of different catalysts was performed at the optimized reaction conditions, and the results are shown in Table 2. Initially, acidic HZSM-5-M shows the highest reaction activity and 1-phenyl-2-(phenylthio)ethanol selectivity, as compared to mesopore-free HZSM-5 and acid-free TS-1-M catalysts (entries 1–4). These results suggest that the catalyst with acidity favors this reaction. Compared with HZSM-5-M, the product selectivity is further improved on the Cu/HZSM-5-M catalyst (entries 2 and 5). The catalytic performance of the Cu/HZSM-5-M catalyst is also superior to that of Fe/HZSM-5-M and Co/HZSM-5-M catalysts (entries 5–7). Notably, although Cu/HZSM-5 catalyst gives similar styrene conversion to that Cu/HZSM-5-M catalyst, the product selectivity over Cu/HZSM-5 is lower than that over Cu/HZSM-5-M (entry 8). This could be due to the difference in porous structure between Cu/HZSM-5 and Cu/HZSM-5-M catalysts. Cu/HZSM-5-M has a high mesoporous surface area of 182 m^2^/g and mesoporous volume of 0.35 cm^3^/g (Table S1). In contrast, Cu/HZSM-5 has only a low external area of 27 m^2^/g. The abundant mesopores in Cu/HZSM-5-M not only could be beneficial to the reactants accessible for active sites, but also favor the diffusion of the reactants, enhancing product selectivity. In addition, the acid-base property of the support strongly influences the reaction activity of their supported Cu catalysts. ETS-10 has strong basicity [13], and the Cu/ETS-10 catalyst only gives styrene conversion of 71% (entry 9), even lower than the reaction system without catalyst, suggesting that the catalyst with basicity inhibits this reaction. Acidic-free TS-1-M supported Cu catalyst (Cu/TS-1-M) presents comparable activity to that Cu/HZSM-5-M, but 1-phenyl-2-(phenylthio)ethanol product selectivity of Cu/TS-1-M (76%) is inferior to that of Cu/HZSM-5-M (84%, entry 10).

Based on our experiment result, it can be concluded that the Cu species and acidic sites on Cu/HZSM-5-M catalyst could enhance the catalytic performance. Our previous work demonstrated that acidic sites on zeolite can promote the adsorption of styrene through interaction of C=C bond with the acidic sites, resulting in the activation of the C=C bond, and the activated C=C bond readily was attacked by electrophilic species to form the desired product [15]. In this work, compared with acid-free TS-1-M supported Cu catalyst, large amounts of acidic sites on Cu/HZSM-5-M catalyst could benefit the adsorption of styrenes, improving the reaction performance in the hydroxysulfurization reaction of styrenes. On the other hand, the Cu^+^ and Cu^2+^ species in the catalyst could play an important role in this reaction. It has been reported that Cu salts acted on the disulfide as a Lewis acid or co-oxidant in air to give electrophilic sulfur species [8]. In our case, Cu^+^ and Cu^2+^ species in the catalyst could also facilitate the formation of electrophilic sulfur species [8], which could attack the styrene to form the target product.

The reaction scope of this reaction over Cu/HZSM-5-M catalyst was also examined, and the results are given in Table 3. Cu/HZSM-5-M catalyst tolerates styrenes with electron-donating (methyl and *t*-butyl) and electron-withdrawing (fluorine, chlorine, and bromine) substituents, affording the desired products in good yields (**3b**–**3i**). The substrate of 2-naphthylethylene can also be employed in this transformation, giving a relatively low yield (**3j**). Furthermore, employing propenyl benzene as a substrate, it affords the desired product **3k** in a gratifying yield. Additionally, diaryl disulfides containing nitro and bromine substituents were smoothly reacted with *p*-methylstyrene, and a moderate yield was obtained with the expected products (**3l** and **3m**). Moreover, using cyclic aliphatic alkene and diaryl disulfides as substrates, the reaction still smoothly proceeded and desired product **3n** in good yield. Notably, when the reagent of 4-pentenoic acid with internal nucleophile was employed as substrate, the target product of 2-((phenylthio)methyl)tetrahydrofuran **3o** also was obtained. These results demonstrated that the Cu/HZSM-5-M catalyst presents good functional group compatibility in hydroxysulfurization reaction.

The reusable ability of Cu/HZSM-5-M catalyst was investigated (Table 4). Cu/HZSM-5-M catalyst exhibits relatively high activity and selectivity after it recycled seventh runs, nevertheless, the activity and product selectivity of Cu/HZSM-5-M were slightly reduced with an increase cycle times. This could be due to the fact that the leaching of Cu species in the Cu/HZSM-5-M catalyst occurred. When the Cu/HZSM-5-M was recycled seven times, the Cu content in the catalyst decreased from 3.0% for fresh one to 2.7 wt. % (Table S1). The reused Cu/HZSM-5-M catalyst was also characterized by TEM, XRD, and N_2_-adsorption. TEM image shows that the metal particles are still present on the reused Cu/HZSM-5-M sample (Figure S4). The characteristic diffraction peaks in XRD pattern of the reused Cu/HZSM-5-M are the same as that of the fresh one (Figure S5). The reused Cu/HZSM-5-M catalyst has high BET surface area (354 m^2^/g), external surface area (175 m^2^/g), and mesoporous volume (0.33 cm^3^/g, Table S1). These results suggest that Cu/HZSM-5-M exhibits good physico-chemical stability.

## 4. Conclusions

In conclusion, hierarchically porous zeolite ZSM-5 with cylinder-like morphology and micro-meso-macroporous structure was synthesized by using cationic copolymer as mesoscale template. After loading metal Cu on the H-form ZSM-5-M, the obtained Cu/HZSM-5-M presents good catalytic performance in the hydroxysulfurization of styrenes with diaryl disulfides, as compared to acidic micropore ZSM-5, acid-free TS-1-M, and basic ETS-10 supported Cu catalysts. This feature should be attributed to the fact that numerous acidic sites and Cu^+^ and Cu^2+^ species in Cu/HZSM-5-M catalyst play a synergistic effect in improving the reaction activity and product selectivity. In addition, the prepared Cu/HZSM-5-M catalyst tolerates a variety of functional groups on both the styrenes and disulphide substrates, and has good reusability.

## Figures and Tables

**Table 1:** Total acidity and acidic site distribution of the HZSM-5-M and HZSM-5 samples ^1^.

Catalyst	Weak Acid Site (µmol·g^−1^)	Medium Acid Site (µmol·g^−1^)	Strong Acid Site (µmol·g^−1^)	Total Acid Site (µmol·g^−1^)
HZSM-5-M	134	71	75	280
HZSM-5	282	110	106	498

^1^ The weak, medium, and strong acid site is estimated from the corresponding area of the deconvoluted peaks, respectively, and the total acid site is determined by acid–base titration.

**Table 2:**
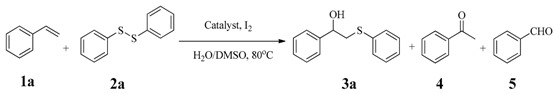
Hydroxysulfurization reaction over different catalysts ^1^.

Entry	Catalysts	Conversion (%) ^3^	Selectivity (%) ^4^		
			3a	4	5
1	-	89	63	24	13
2	HZSM-5-M	91	79	15	16
3	TS-1-M	90	70	16	14
4	HZSM-5	89	68	14	18
5	Cu/HZSM-5-M	92	85	10	5
6	Fe/HZSM-5-M ^2^	89	65	21	14
7	Co/HZSM-5-M ^2^	82	64	16	20
8	Cu/HZSM-5	89	70	17	13
9	Cu/ETS-10	71	66	22	12
10	Cu/TS-1-M	91	76	15	9

^1^ Reaction condition: 25 mg solid catalyst, alkenes (1.0 mmol), diaryl disulfide (0.6 mmol), oxidant (0.2 mmol), H_2_O (1 mL), DMSO (1 mL), 80 °C for 10 h. ^2^ The metal loading of Fe and Co. on Fe/HZSM-5-M and Co/HZSM-5-M catalysts is 3.0 wt. %. ^3,4^ The conversion and selectivity were analyzed by an Agilent 7890B GC (Agilent Technologies Inc., Santa Clara, CA, USA) equipped with a flame ionization detector.

**Table 3:** Hydroxysulfurization reaction between alkenes and diaryl disulfides ^1^.

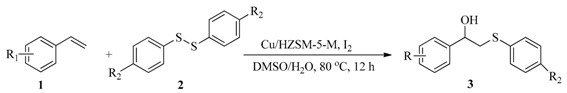
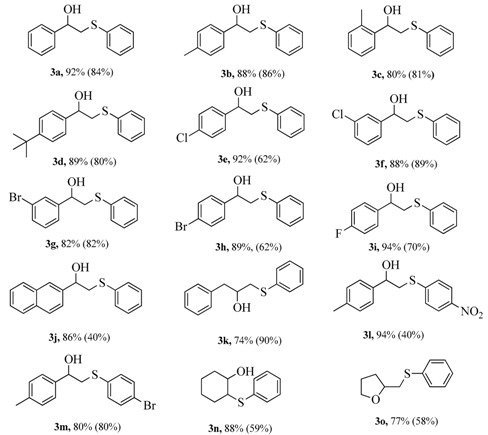

^1^ Reaction condition: 25 mg solid catalyst, alkenes (1.0 mmol), diaryl disulfide (0.6 mmol), I_2_ (0.2 mmol), H_2_O (1.0 mL), DMSO (1.0 mL), 80 °C for 10 h. The data out of parenthesis is conversion, and in parenthesis is selectivity, and the conversion and selectivity were analyzed by an Agilent 7890B GC equipped with a flame ionization detector.

**Table 4:**
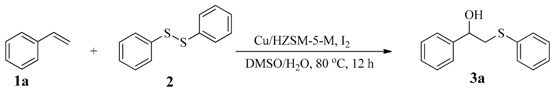
The reusability of the catalyst ^1^.

Entry	Recycle	Conversion (%)	Selectivity (%)
1	Run 1	92	84
2	Run 2	92	84
3	Run 3	91	83
4	Run 4	90	82
5	Run 5	89	82
6	Run 6	87	80
7	Run 7	88	80

^1^ Reaction condition: 25 mg solid catalyst, styrene (1.0 mmol), diaryl disulfide (0.6 mmol), I_2_ (0.2 mmol), H_2_O (1.0 mL), DMSO (1.0 mL), reaction time of 10 h. The yield was analyzed by GC. The spent catalyst was carefully collected, washed with acetonitrile 10 times, and dried at 120 °C for 10 h, and followed by calcination at 450 °C in air for the next recycle.

## Supplementary Materials

The following are available online at http://www.mdpi.com/2079-4991/7/12/459/s1, Figure S1: XRD patterns of (a) TS-1-M and Cu/TS-1-M and (b) ETS-10 and Cu/ETS-10 samples; Figure S2: The pore size distributions of the (■) ZSM-5-M and (●) Cu/HZSM-5-M samples; Figure S3: SEM images of the (a) TS-1-M, (b) ZSM-5 and (c) ETS-10 zeolites; Figure S4: TEM image of reused Cu/HZSM-5-M catalyst; Figure S5: XRD patterns of fresh and reused Cu/HZSM-5-M catalysts; Table S1: Texture parameters of the different samples; Table S2: The results of deconvolution of XPS Cu2p3/2 peak of Cu/HZSM-5-M and Cu/TS-1-M samples; Table S3: Optimization of reaction conditions.
