# Supplementary material for: Acidic Mesoporous Zeolite ZSM-5 Supported Cu Catalyst with Good Catalytic Performance in the Hydroxysulfurization of Styrenes with Disulfides
Source: Nanomaterials (Basel). 2017 Dec 19;7(12):459. doi: 10.3390/nano7120459 (PMC5746948; doi:10.3390/nano7120459)
Supplement: Supplementary file 1 [file nanomaterials-07-00459-s001.pdf]

Supporting Information

Acidic Mesoporous Zeolite ZSM-5 Supported Cu Catalyst with Good Catalytic Performance in the Hydroxysulfurization of Styrenes with Disulfides

Jun Hu[1], Chaojie Zhu[2], Feifei Xia[1], Zhongxue Fang[2], Fengli Yang[1], Jushi Weng[1], Pengfei Yao[1], Chunzhi Zheng[1], Hai Dong[2], Wenqian Fu[2*]

[1] School of Chemical and Environmental Engineering, Jiangsu University of Technology, Changzhou, Jiangsu 213001, China.

[2] School of Petrochemical Engineering, Changzhou University, Changzhou, Jiangsu 213164, China.

[*] Correspondence: fuwenqian@cczu.edu.com, phone: +86-0519-8633-0009

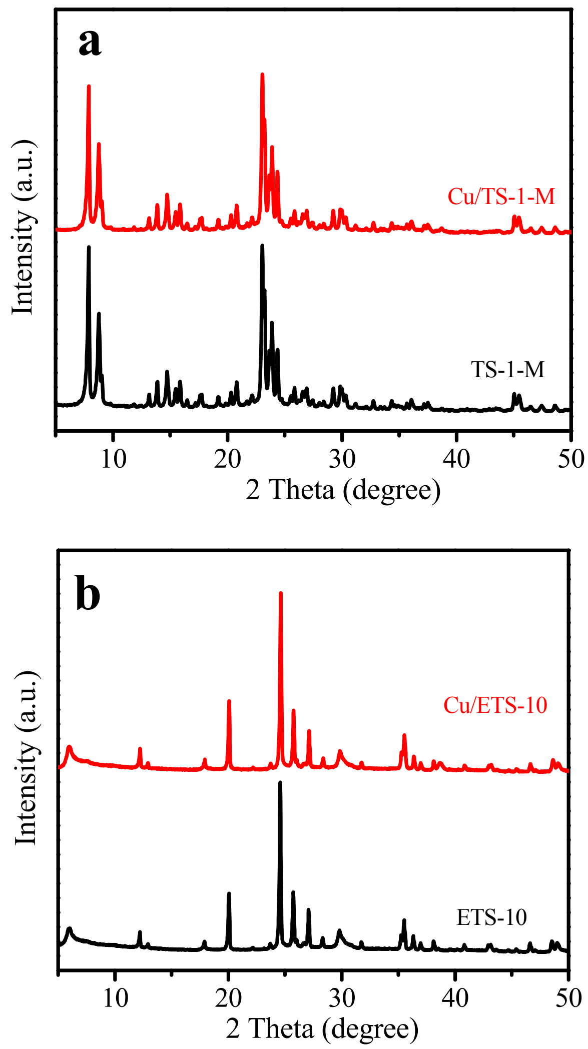

Figure S1. XRD patterns of (a) TS-1-M and Cu/TS-1-M and (b) ETS-10 and Cu/ETS-10 samples.

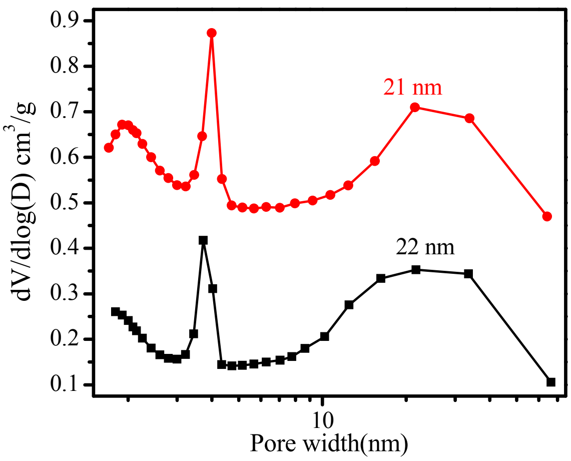

Figure S2. The pore size distributions of the (■) ZSM-5-M and (●) Cu/HZSM-5-M samples.

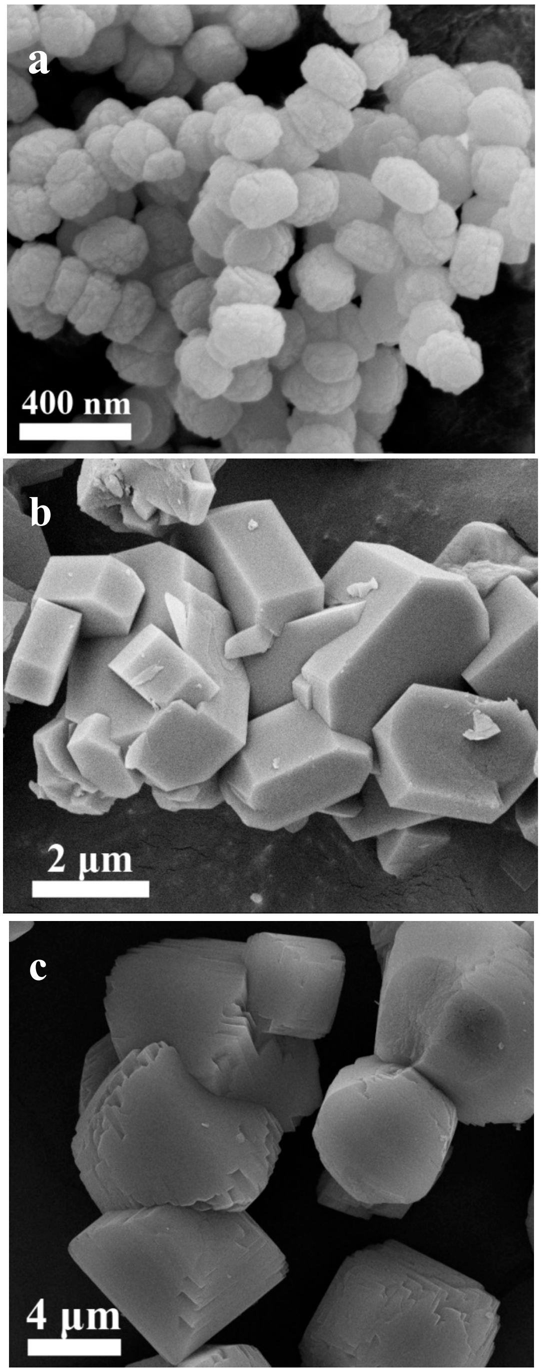

Figure S3. SEM images of the (a) TS-1-M, (b) ZSM-5 and (c) ETS-10 zeolites.

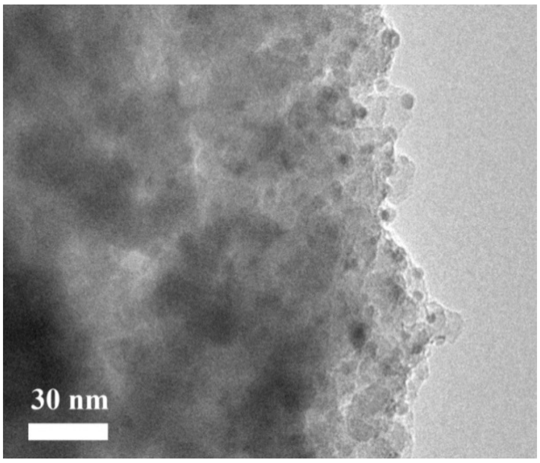

Figure S4. TEM image of reused Cu/HZSM-5-M catalyst.

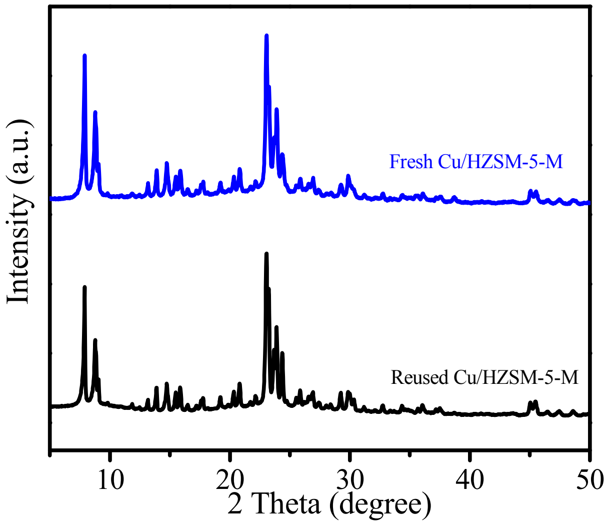

Figure S5. XRD patterns of fresh and reused Cu/HZSM-5-M catalysts.

Table S1 Texture parameters of the different samples.

Sample	S_{BET} (m^2/g)[1]	$S_{ext.}$ (m^2/g)[2]	$V_{mic.}$ (cm^3/g)[3]	$V_{meso.}$ (cm^3/g)[4]	Cu (wt.%)[5]	Si/Al(Ti)[5]
ZSM-5-M	432	195	0.10	0.45	-	26
TS-1-M	536	210	0.12	0.25	-	-
ZSM-5	304	31	0.11	0.02	-	20
ETS-10	311	30	0.12	0.02	-	4.5
Cu/HZSM-5-M	360	182	0.07	0.35	3.0	-
Cu/HZSM-5	300	27	0.09	0.02	2.9	-
Cu/TS-1-M	469	180	0.11	0.22	3.0	-
Cu/ETS-10	307	23	0.11	0.03	2.9	-
Reused Cu/HZSM-5-M	354	172	0.07	0.33	2.7	-

[1] BET surface area. [2] External surface area, including mesoporous surface area. [3]Microporous volume. [4]Mesoporous volume. [5]The Cu content and the atomic Si/Al(Ti) ratio in the different catalyst was determined by ICP.

Table S2 The results of deconvolution of XPS Cu2p$_{3/2}$ peak of Cu/HZSM-5-M and Cu/TS-1-M samples.

Catalysts	Binding energy of Cu 2p$_{3/2}$(eV)		Cu content (at.%)	
	Cu^+	Cu^{2+}	Cu^+	Cu^{2+}
Cu/HZSM-5-M	933	935.3	55	45
Cu/TS-1-M	933.5	936.1	79	21

Table S3 Optimization of reaction conditions.[1]

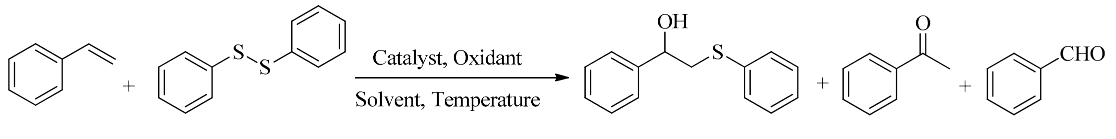

Entry	Oxidant	Solvent	T.[2](°C)	Conversion[3] (%)	Selectivity[4] (%)		
					**3a**	**4**	**5**
1	I_2	DMSO/H_2O	80	89	63	24	13
2	I_2	CH_2Cl_2/H_2O	80	67	54	35	11
3	I_2	THF/H_2O	80	59	51	27	22
4	I_2	1,4-Dioxane/H_2O	80	76	49	35	16
5	I_2	CH_3CN/H_2O	80	83	57	28	15
6	I_2	Toluene/H_2O	80	86	60	24	16
7	TBHP	DMSO/H_2O	80	24	62	23	15
8	H_2O_2	DMSO/H_2O	80	18	58	21	21
9	O_2	DMSO/H_2O	80	15	-	10	90
10	$K_2S_2O_8$	DMSO/H_2O	80	16	-	23	77
11	$KMnO_4$	DMSO/H_2O	80	21	-	34	66
12	I_2	DMSO/H_2O	90	89	62	24	14
13	I_2	DMSO/H_2O	70	77	63	24	13
14	I_2	DMSO/H_2O	60	67	60	25	15
15	I_2	DMSO/H_2O	50	-	-	-	-

[1]Reaction condition: alkenes (1.0 mmol), diaryl disulfide (0.6 mmol), Oxidant (0.2 mmol), H_2O (1.0 mL), Solvent (1 mL), 80 °C for 10 h. [2]T.: Temperature. [3,4]The conversion and product selectivity were analyzed by gas chromatography.
